# 4-1BB: A promising target for cancer immunotherapy

**DOI:** 10.3389/fonc.2022.968360

**Published:** 2022-09-14

**Authors:** Alyssa Min Jung Kim, Macy Rose Nemeth, Seung-Oe Lim

**Affiliations:** ^1^ Department of Medicinal Chemistry and Molecular Pharmacology, Purdue University, West Lafayette, IN, United States; ^2^ Purdue Institute of Drug Discovery, Purdue University, West Lafayette, IN, United States; ^3^ Purdue Center for Cancer Research, Purdue University, West Lafayette, IN, United States

**Keywords:** 4-1BB, CD137, cancer, immunotherapy, therapeutic antibody, posttranslational modification

## Abstract

Immunotherapy, powered by its relative efficacy and safety, has become a prominent therapeutic strategy utilized in the treatment of a wide range of diseases, including cancer. Within this class of therapeutics, there is a variety of drug types such as immune checkpoint blockade therapies, vaccines, and T cell transfer therapies that serve the purpose of harnessing the body’s immune system to combat disease. Of these different types, immune checkpoint blockades that target coinhibitory receptors, which dampen the body’s immune response, have been widely studied and established in clinic. In contrast, however, there remains room for the development and improvement of therapeutics that target costimulatory receptors and enhance the immune response against tumors, one of which being the 4-1BB (CD137/ILA/TNFRSF9) receptor. 4-1BB has been garnering attention as a promising therapeutic target in the setting of cancer, amongst other diseases, due to its broad expression profile and ability to stimulate various signaling pathways involved in the generation of a potent immune response. Since its discovery and demonstration of potential as a clinical target, major progress has been made in the knowledge of 4-1BB and the development of clinical therapeutics that target it. Thus, we seek to summarize and provide a comprehensive update and outlook on those advancements in the context of cancer and immunotherapy.

## Introduction

Since its emergence, immunotherapy, as a rapidly evolving area in the field of oncology, has become a prominent and promising means of therapy in various types of cancer. Whilst encompassing a broad range of treatment agents such as immune checkpoint blockade therapies, vaccines, and adoptive cell therapies, strategies largely focused on harnessing the body’s immune system by modulating coinhibitory and costimulatory receptors expressed on immune cells (1). These coinhibitory and costimulatory receptors, or immune checkpoints, are primarily expressed on T cells and function in regulating and finetuning the body’s immune response when stimulated (1). Thus, their vital role in modulating the immune system and the ability of cancer cells to alter the function and expression of these receptors make them attractive therapeutic targets. With the majority of approved immune checkpoint therapies directed towards blocking coinhibitory receptors such as cytotoxic T lymphocyte-associated molecule-4 (CTLA-4), programmed cell death receptor-1 (PD-1), and programmed cell death ligand-1(PD-L1) to revitalize the immune response against tumors, there remains room for the development, improvement, and clinical application of therapeutics that target costimulatory receptors capable of enhancing the immune response (1, 2). Here, we seek to review one of the costimulatory receptors, 4-1BB (CD137/ILA/TNFRSF9), and the progress made, trends, and future outlooks in the development of therapeutics that target it.

Stimulation of 4-1BB, a surface receptor expressed on activated T cells amongst many other types of immune cells, activates various signaling pathways involved in the generation of a potent cellular immune response. Thus, its ability to modulate a wide range of immune cells and, in particular, its role in mediating T cell survival, proliferation, and effector function, have made it an attractive target, amongst other costimulatory molecules such as OX40, GITR, and CD27, in the development of cancer immunotherapy (3, 4). This clinical promise and value of 4-1BB are reflected in the continuous advancements and growth in the knowledge of the receptor as well as the breadth of therapeutic strategies that have gone through or are currently undergoing pre-clinical and clinical trials. Thus, we provide a comprehensive review and a greatly needed update of the receptor, 4-1BB, and its role in disease and immunotherapy. We will discuss the recent progresses that have been made and the current trends in the development of therapeutics targeting the receptor, concluding with potential strategies and outlooks for future research.

## 4-1BB expression and signaling

### History

4-1BB is a costimulatory glycoprotein receptor that is part of the tumor necrosis factor superfamily (TNFRSF). It was initially discovered by Kwon et al. in 1989 as a receptor protein expressed on activated cytolytic and helper T lymphocytes (5). Following studies conducted by Pollok et al. further characterized 4-1BB, revealing that the protein is an inducible cell surface receptor that is expressed in the presence of activating stimuli and functions in cell signaling during T cell activation and proliferation (6). The endogenous ligand for 4-1BB, 4-1BBL (CD137L/TNFSF9), was identified by Goodwin et al. subsequently, where they identified that the interaction of the receptor with its ligand results in thymocyte and splenic T cell proliferation, suggesting the role of the 4-1BB/4-1BBL interaction to be a part of the body’s immune response (7). Later, Hurtado et al. demonstrated that T cell proliferation and activation mediated by the 4-1BB/4-1BBL interaction could be blocked by antibodies that target the receptor and prevent 4-1BBL binding, suggesting a potential area for immunotherapy development (8). Additional studies further indicated that antibodies directed against 4-1BB are also capable of providing costimulatory signals to the T cell, reproducing the agonism provided by the endogenous 4-1BBL (9).

Subsequent studies displayed that 4-1BB expression was not solely limited to T lymphocytes. Instead, 4-1BB was shown to also be expressed on other immune cells such as monocytes, dendritic cells (DCs), and natural killer (NK) cells (10–12). In addition, its expression profile was identified to be even more extensive as 4-1BB was also observed on epithelial and endothelial cells such as adipocytes, vascular endothelial cells, skeletal muscle cells, and malignant cells (13–16). The receptor’s natural ligand, 4-1BBL, was shown to be mainly expressed on professional antigen presenting cells (APCs), including DCs, macrophages, and B cells (7, 17). The findings that the 4-1BB/4-1BBL axis stimulates the effector functions of T cells and is expressed on a wide variety of cell populations together point to the clinical potential of utilizing the strategy of blocking or stimulating the signaling pathway in the treatment of immune disorders as well as cancer.

### 4-1BB signaling in different cell types

As previously mentioned, the expression profile of 4-1BB encompasses a variety of cell types in which they may have different functions and means of expression. Nonetheless, on all cells it is expressed on, 4-1BB stimulation results in the activation of multiple signaling pathways downstream of the receptor. Currently, the only known intercellular ligand of the 4-1BB receptor is 4-1BBL (18). 4-1BBL expression is induced in activated APCs and is also seen in myeloid progenitor cells and hematopoietic stem cells (11). Expressed on professional APCs, including macrophages, DCs, and activated B cells, as well as on nonhematopoietic cells, such as fibroblasts, endothelial cells, and epithelial cells, the crosslinking of trimerized 4-1BBL activates 4-1BB (18, 19). Of note, studies have shown that mouse 4-1BB can also bind to ubiquitous extracellular membrane (ECM) proteins such as fibronectin, vitronectin, laminin, and collagen VI (20, 21). However, such interaction between 4-1BB and ECM proteins was shown to not be conserved in humans (21, 22).

4-1BB, a type I transmembrane glycoprotein, has an extracellular domain made up of four cysteine-rich domains (CRDs), a helical transmembrane domain, and a cytoplasmic signaling domain (23). The extracellular domain of 4-1BBL, a type II transmembrane protein, is a homotrimer of an extended, three-bladed propeller structure (24). Upon interaction, the 4-1BB/4-1BBL complex assumes a structure that is similar to that of established TNF receptor-ligand complexes where the ligand trimer takes on the canonical bell shape and the three monomeric receptors are positioned around the exterior face of each protomer in parallel to the ligand trimer, ultimately forming a hetero-hexamer signaling axis (23, 25).

Upon interaction with 4-1BBL and subsequent activation, 4-1BB signaling is primarily mediated by the recruitment of TNFR-associated factors 1 and 2 (TRAF1 and TRAF2) to the TRAF-binding motif located in the 4-1BB cytoplasmic tail (26–29). The TRAF proteins form homo- or hetero-trimers and serve as scaffold proteins, activating the downstream effectors of various signaling cascades leading to the different cellular responses (18, 30). While TRAF1 and 2 have been well-established to be part of the 4-1BB signalosome, TRAF3 has only been suggested to potentially be a part of it, notably in 4-1BB-based chimeric antigen receptor (CAR) T cells, yet further research is warranted to determine their presence in other cell types (18, 31).

**Figure 1:**
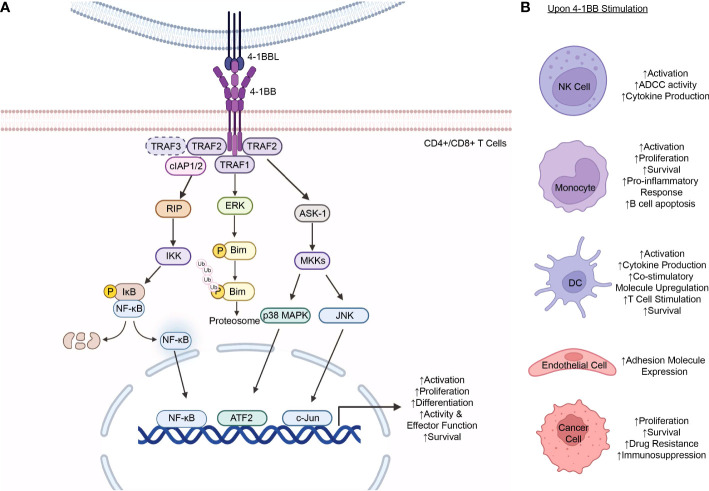
4-1BB Expression and Signaling. **(A)** 4-1BB stimulation on T cells results in the activation of multiple signaling pathways downstream the receptor. Upon interaction with 4-1BBL and subsequent activation, 4-1BB signaling is initially mediated by the recruitment of TRAF1, TRAF2, or, hypothetically, TRAF3 (in dashed lines), to the TRAF-binding motif located in the 4-1BB cytoplasmic tail. The TRAF proteins form a homo- or hetero-trimer and, in turn, recruits cIAP1/2 which further mediates the activation of downstream effectors that transduce signals down various signaling cascades to the nucleus including the NF-κB, ERK, p38 MAPK, and JNK pathways. Signaling down these pathways results in the increased expression of the anti-apoptotic proteins, Bfl-1 and Bcl-x_L_, decreased expression of the pro-apoptotic protein Bim, and increased proliferation, differentiation, effector functions, and survival of the T cells. **(B)** 4-1BB expression is observed in a wide range of cells including NK cells (4, 35), monocytes (36–38), DCs (11, 39, 40), endothelial cells (14, 41), and malignant cancer cells (16, 42–44). Stimulation of 4-1BB on the respective cells result in varying cellular responses depending on the cell type. TRAF, tumor necrosis factor receptor-associated factor; cIAP, cellular inhibitor of apoptosis protein; RIP, receptor interacting protein; IKK, IκB kinase; IκB, inhibitor of NF- κB; NF- κB, nuclear factor-kappa B; ERK, Extracellular Signal-Regulated Kinase; Bim, Bcl-2-like protein 11; ASK-1, apoptosis signal-regulating kinase 1; MKK, Mitogen-Activated Protein Kinase Kinase; MAPK, mitogen-activated protein kinases; JNK, Jun N-terminal kinase; ATF2, activating transcription factor 2; P, phosphate; Ub, ubiquitin; NK cell, natural killer cell; ADCC, antibody-dependent cellular cytotoxicity; DC, dendritic cell; Bfl-1, Bcl-2-related protein A1; Bcl-x_L_, B cell lymphoma-extra large. The figure was created with BioRender.com.

Most 4-1BB expression is transient and activation induced as seen in activated T and NK cells. On T cells, while resting T cells do not express 4-1BB, its expression is transiently induced upon T cell receptor (TCR) stimulation and CD3 signaling (32–34). Following expression and ligand binding, the 4-1BB/4-1BBL interaction delivers a costimulatory signal mediated through the TRAF1 and TRAF2 trimers, which recruit the ubiquitin ligases, cellular inhibitors of apoptosis 1 or 2 (cIAP1/2), that activate the downstream signaling pathways: the nuclear factor kappa B (NF-κB), extracellular signal regulated kinase (ERK), p38 mitogen-activated protein kinase (MAPK), and c-Jun N-terminal kinase (JNK) pathways (**Figure 1A**) (18, 30, 34). The activation of these pathways results in the costimulatory signals that further augment CD4^+^ and CD8^+^ T cell proliferation, differentiation, and effector functions (4, 45, 46). Furthermore, 4-1BB signaling has been shown to induce the expression of the anti-apoptotic proteins, Bcl-2-related protein A1 (Bfl-1) and B cell lymphoma-extra large (Bcl-x_L_), mediated by the transcription factor NF-κB, in CD8^+^ T cells, enhancing cell survival (30, 47, 48). In addition, another mechanism of 4-1BB signaling that promotes T cell survival involves the downregulation of the pro-apoptotic Bcl-2-like protein 11 (Bim), which is downstream of the ERK pathway (48). Of note, it has been shown that such costimulatory function of 4-1BB signaling involved in the activation of T cells is preferentially observed in CD8^+^ T cells compared to CD4^+^ T cells (46).

As aforementioned, 4-1BB expression is not limited to T cells and is observed in a wide range of cell types such as NK cells, monocytes, DCs, endothelial cells, and cancer cells, and cellular responses to stimulation vary depending on the cell (**Figure 1B**) (29). Unlike T cells, NK cells do not express the TCR complex, but, instead, express a range of activating and inhibitory receptors on the cell surface that enable them to recognize their target cells as well as regulate their functions. Amongst these regulatory molecules are those of the TNF/TNFR family (49). Of those, the expression of 4-1BB is upregulated in the presence of immunoglobulins caused by the Fc cross-linking of the FcγRIII Fc receptor (CD16) on the NK cells (29, 50). Upon expression and ligand binding, 4-1BB signaling, mediated by 4-1BBL expressing cells, enhances NK cell proliferation as well as the antibody-dependent cell-mediated cytotoxicity (ADCC) function of the cells following activation with 4-1BB antibodies (4, 29, 35). Furthermore, 4-1BB signaling on NK cells also results in increased cytokine production, including IFN-γ, which, in turn, stimulates T cells, particularly CD8^+^ T cells, prompting increased proliferation, cytokine production, and activity of the cells (35).

4-1BB expression on monocytes was shown to be induced upon stimulation with proinflammatory mediators (10). Regarding its role in monocytes, 4-1BB signaling has been shown to activate the cells as well as enhance their proliferation and survival (36, 37). Kienzle et al. further demonstrated that upon stimulation with its ligand and subsequent activation of the monocytes, 4-1BB signaling produces a proinflammatory response, increasing TNF-α and IL-8 whilst decreasing IL-10 secretion (38). Furthermore, 4-1BB activation and signaling on monocytes exhibited the ability to induce apoptosis of B cells mediated by cell-cell contact between other cell surface molecules and, potentially, the cytokines, TNF-α and IFN-γ, produced by monocytes upon activation (38).

While most 4-1BB expression is activation induced, exceptions include its expression in DCs on which the receptor is expressed constitutively (51). This constitutive expression of the receptor protein on DCs was initially discovered by Futagawa et al. where they found that such expression was also at a high level on this class of APCs. This was, in turn, downregulated with the stimulation of CD40, another receptor of the TNFRSF (11). 4-1BB signaling on these cells led to their activation, subsequent IL-6 and IL-12 cytokine production, and upregulation of the costimulatory molecules CD80 (B7-1) and CD86 (B7-2) (11, 39). Furthermore, Wilcox et al. demonstrated that activation of 4-1BB signaling expressed on DCs achieved by the administration of an agonistic 4-1BB antibody may also augment their roles in stimulating a T cell response (39). Lastly, 4-1BB expression on DCs was shown to have a vital role in DC function and survival with 4-1BB^-/-^ mice having altered survival in the studies completed by Choi et al. (40).

4-1BB expression is also inducible on nonhematopoietic cells, including endothelial and epithelial cells (19). Drenkard et al. demonstrated that 4-1BB expression is induced by proinflammatory cytokines in vascular endothelial cells especially at sites of inflammation (41). Such expression and 4-1BB signaling, in turn, was shown to upregulate expression of the adhesion molecules, vascular cell adhesion molecule‐1 (VCAM-1) and intercellular adhesion molecule‐1 (ICAM-1), which facilitate peripheral monocyte migration from the bloodstream into the sites of inflammation (14, 41).

4-1BB has also been detected on various types of malignant cells, including lung cancer, leukemia, lymphoma, and pancreatic cancer (16, 42–44). At present, the regulatory mechanisms of its expression and its biological significance in cancer cells remain largely unknown. Nonetheless, Glorieux et al. suggested the potential role of the oncogenic K-ras and its downstream MAPK and NF-κB signaling pathways in the induction of 4-1BB expression in pancreatic cancer cells (16). In hematologic malignancies, it has been shown that the expression and signaling of 4-1BB and its ligand, 4-1BBL, on T and B leukemia cells enhance the proliferation and survival of the malignant cells as well as promote drug resistance (43).

## Posttranslational modifications (PTMs) of 4-1BB

Recent studies have indicated the regulatory role of posttranslational modifications (PTMs), specifically glycosylation and ubiquitination, in 4-1BB expression (52, 53). Glycosylation, the covalent conjugation of carbohydrate chains, at two different sites (N138 and N149) of the 4-1BB protein was shown to have a role in the maturation and localization of the protein to the cell membrane, with its absence preventing 4-1BB membrane expression (53). Of note, glycosylation, including *N*-linked glycosylation, the covalent attachment of an N-acetylglucosamine (GlcNAc) to the nitrogen atom on the side chain of an asparagine (Asn/N) residue *via* an N-glycosidic bond, has been shown to have a contributory role in the immune system, especially as means of intracellular regulation of immune receptor and ligand proteins (54).

**Figure 2:**
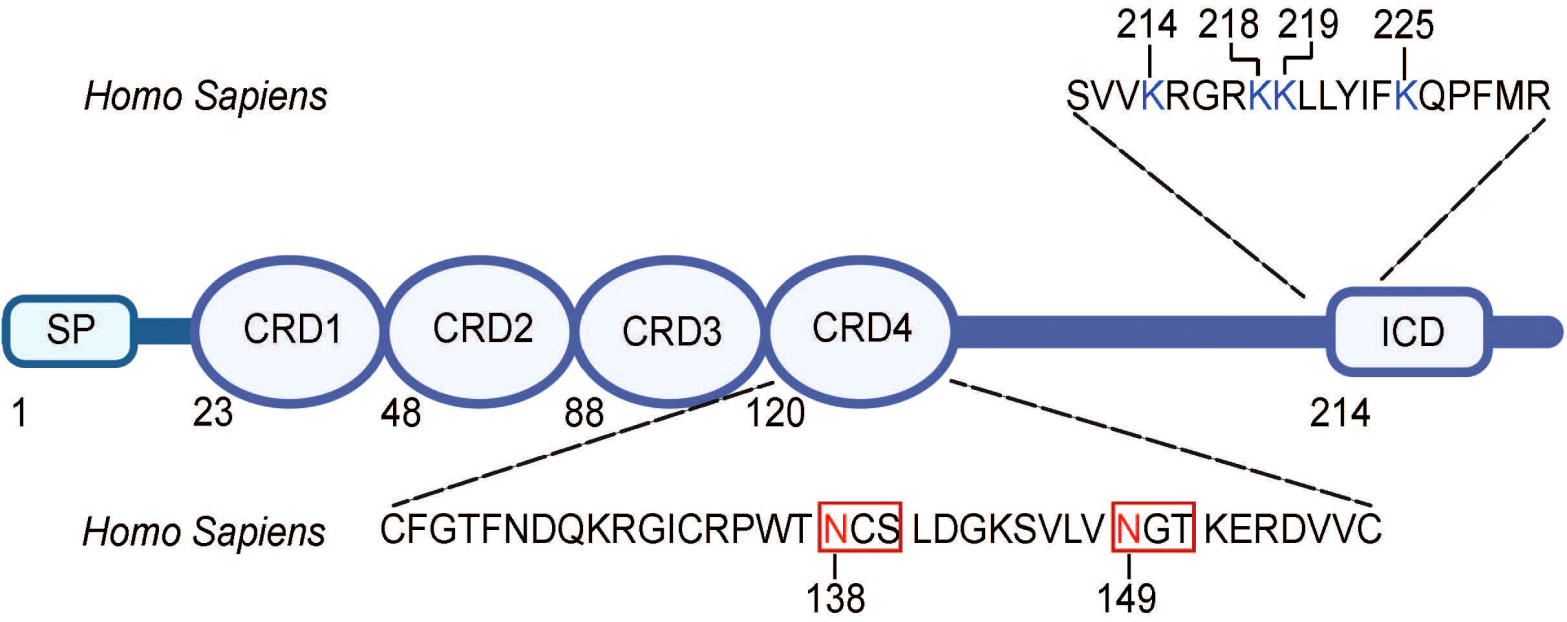
Amino acid sequence for sites of N-glycosylation and ubiquitination in human 4-1BB. *N-*linked glycosylation N-X-S/T motifs are found in the extracellular domain (CRD4) of 4-1BB and are boxed. Asparagine (N) residues that are glycosylated (N138, N149) is highlighted in red. The lysine resides that are ubiquitinated are found in the intracellular domain of 4-1BB and are highlighted in blue. SP, signal peptide; CRD, cysteine-rich domain; ICD, intracellular domain. The figure was created with BioRender.com.

With regard to 4-1BB, its two *N*-linked glycosylation sites (N138, N149) were determined to be located away from the receptor-ligand interface, on N-X-S/T motifs, the motif where canonical *N*-glycosylation occurs, in CRD4, suggesting that it did not function in mediating the 4-1BB/4-1BBL interaction (**Figure 2**) (25, 54). Despite having been long known to be heavily modified by glycosylation, little was known about the biological importance of this modification on the receptor. However, recently, Sun et al. elucidated the role of this glycosylation, determining that it facilitates 4-1BB membrane localization by preventing its multimerization intracellularly and, thus, permitting its membrane transportation and fast turnover (53). With the absence of the *N*-glycans, the “under-glycosylated” proteins form stable dimers and oligomers, which, consequently, hinders the trafficking of the proteins to the cell membrane (53). Of note, glycosylation patterns have been observed to be altered in presence of disease, including cancer cells, which, consequently, impact the expression of proteins, including cell membrane receptors (55). This difference in glycosylation moieties and profiles may, perhaps, serve as a basis for future therapeutic development.

Furthermore, in terms of ubiquitination, 4-1BB has been found to have four different sites of ubiquitination on its intracellular domain (K214, K218, K219, K225) (**Figure 2**) (52). Sun et al., thus, derived that 4-1BB is degraded and cleared *via* the ubiquitin-proteosome system (UPS) and such degradation can be slowed with the administration of a proteosome inhibitor or insertion of mutations at the ubiquitination site residues (52). The prolonged expression of 4-1BB at the cell surface, mediated by these strategies, allowed for augmented signaling down the receptor and transcription of the downstream genes. In addition, *N*-glycosylation of mature 4-1BB was observed to cause the receptor to be more prone to ubiquitination-mediated degradation, suggesting an interplay between the glycosylation and ubiquitination modifications of the protein (52). This suggests room for clinical intervention, such as treatment with proteasome inhibitors or inhibitors of enzymes involved in the ubiquitination and degradation processes, to prolong 4-1BB expression and signaling. Altogether, these findings indicate the potential of the glycosylation or ubiquitination status of 4-1BB as predictors of response to 4-1BB targeted therapy as well as their modulation as a clinical strategy in the treatment of diseases.

## 4-1BB in cancer and non-cancer diseases

With its broad expression profile, 4-1BB signaling have been shown to have a significant role in the body’s immune modulation. This was demonstrated in studies by Kwon et al. where T cells of 4-1BB deficient mice maintained their proliferative ability in response to stimulation, yet failed to secrete cytokines and exert their cytotoxic T lymphocyte activities (56). Thus, with its expression in diverse cell types and biological significance, 4-1BB has further been shown to be involved in a wide variety of pathologies. Although largely known as a costimulatory molecule functioning in the activation of the immune response, 4-1BB signaling was also observed to be involved in its modulation, as in autoimmune diseases, as well as in driving diseases, as in cancer, depending on pathology, indicating that the essence of its activity may be cell-, tissue-, environment- dependent.

The anti-tumor activity of 4-1BB signaling has long been established. Melero et al. initially demonstrated the anti-tumoral potential of treatment with 4-1BB antibodies in murine P815 mastocytoma and Ag104A sarcoma, where it was displayed to be mediated by the amplified proliferation and effector functions of tumor-specific cytotoxic T cells induced by 4-1BB signaling upon antibody binding (57). Such anti-tumoral activity of 4-1BB was also detected in models of lymphoma and hepatocellular carcinoma (58, 59). Additionally, anti-tumoral activity of 4-1BB signaling was shown to be correlated with enhanced survival in a study done by Ju et al. where 4-1BB-intact mice demonstrated longer survival compared to 4-1BB-deficient mice in a melanoma model (60). Furthermore, loss-of-function mutations in the *TNFRSF9* gene, which encodes for 4-1BB, have been reported to result in recurrent infections as well as an Epstein-Barr virus (EBV)-induced lymphoproliferation of both T and B cells (61, 62).

4-1BB signaling, as can be implied from its importance in modulating immunity, has been revealed to augment the body’s immune response against bacterial, fungal, and viral infections as well. Multiple studies have demonstrated that stimulating 4-1BB signaling results in the anti-pathogenic immune responses against infections, including those of *Streptococcus pneumoniae*, *Listeria monocytogenes*, *Candida albicans*, influenza virus, hepatitis C virus, and cytomegalovirus (63–67).

Of note, although known for its immunostimulatory effects as seen in the prior examples, 4-1BB signaling has also been shown to be involved in mitigating various autoimmune diseases. Agonistic 4-1BB antibody treatment demonstrated to be effective in alleviating experimental autoimmune encephalomyelitis, an experimental model of human multiple sclerosis, in which the increased secretion of IFN-γ by CD8^+^ T cells, in turn, induced the expression of indoleamine-2,3-dioxygenase (IDO), an immunosuppressive immune regulator, on APCs (68). A similar mechanism against autoimmunity was also observed in models of rheumatoid arthritis (69). In addition, 4-1BB signaling exhibited protective activities against systemic lupus erythematosus-like autoimmune disease in mice, with its deletion exacerbating the disease (70). Stimulation of 4-1BB signaling mediated by a 4-1BB antibody has also been reported to exert preventative effects on the development of autoimmune type 1 diabetes in non-obese diabetic (NOD) mice (71).

As a prominent mediator of immune responses expressed on various cell types, 4-1BB signaling not only exerts protective effects, but is also capable of driving pathologies such as the adverse effects observed following administration of therapeutic 4-1BB antibodies. The persistent stimulation of 4-1BB signaling and, consequently, continuous activation of T cells have been shown to result in granuloma formation in tumor-draining lymph nodes due to the excessive recruitment of macrophages (72). Furthermore, liver-associated toxicity has been reported to be a common problem associated with therapeutic 4-1BB antibody treatment. Dubrot et al. showed that 4-1BB antibody treatment results in CD8^+^ T cell infiltration into the liver causing inflammation and increased transaminase expression (73). Such infiltration, however, was not associated with clinical benefit in the setting of tumors in or around the liver tissue (73). In addition, activation of liver-resident myeloid cells through the 4-1BB signaling pathway has been shown to cause the production of IL-27, which is also associated with CD8^+^ T cell infiltration, increase in transaminase, and consequent hepatitis (74).

All in all, it can be concluded that 4-1BB expression and signaling has a significant role as a costimulatory immune receptor, whether it be therapeutic or pathogenic. Its wide expression profile and ability to harness the immune system mediated by its downstream signaling demonstrates the clinical promise, as well as challenges, it presents in the treatment of many diseases, including cancer. As can be implied, many efforts have been made in the development of immunotherapies targeting 4-1BB as well as in the strategies to avoid the off-target adverse effects and toxicities associated with 4-1BB stimulation.

## 4-1BB in cancer immunotherapy

The ability of 4-1BB signaling to induce a potent anti-tumoral immune response makes it a promising candidate in the development of cancer immunotherapies. As previously discussed, early studies of 4-1BB as a therapeutic target in cancers were performed by Melero et al. in 1997 where they demonstrated the clinical potential of 4-1BB agonism (57). Treatment with 4-1BB monoclonal antibodies was shown to be capable of eliciting profound tumor suppression mediated by the augmented T cell response subsequent of 4-1BB stimulation in both poorly- and highly- immunogenic murine tumor models (57). Many studies that followed further demonstrated the anti-tumor activity and promise of 4-1BB agonism, alone or in combination with other therapeutic strategies, in different types of cancer, including lymphoma, hepatocellular carcinoma, and melanoma (58, 59).

Briefly, the mechanism of 4-1BB agonism in the treatment of cancer is believed to be dependent on and mediated by the activation of multiple anti-tumoral immune pathways, primarily in CD8^+^ T cells that infiltrate the tumors. The agonistic antibodies are perceived to stimulate the CD8^+^ T cells and promote their proliferation, survival, and cytolytic activity including increased cytokine production, such as IFN-γ, ultimately leading to tumor regression (46). Of note, Martinez-Forero et al. determined that 4-1BB agonistic antibodies, upon binding to the receptor, induce 4-1BB and antibody/ligand complex internalization into an endosomal compartment where downstream signaling continues (75). Furthermore, it has been shown that secretion of perforin and granzymes and activation of the Fas/Fas Ligand (FasL) signaling pathway, a pathway that regulates apoptosis, are other potential cytolytic mechanisms of CD8^+^ T cells induced by 4-1BB agonism (76). 4-1BB stimulation with agonistic antibodies has also been observed to act on 4-1BB expressed on monocytes and macrophages where it induces metabolic and functional reprogramming of the respective cells, promoting their anti-tumor activities (77). In addition, it has been demonstrated that 4-1BB signaling also enhances the metabolic and mitochondrial functions of T cells, promoting their longevity and, consequently, a longer anti-tumor immune response following antibody treatment (78, 79).

Currently, there are two leading 4-1BB agonistic monoclonal antibodies (mAbs) under clinical investigation: urelumab (BMS-663513) and utomilumab (PF-05082566) (80, 81). Urelumab, a non-ligand-blocking fully human IgG4 mAb, was the first of the two to undergo clinical trials as monotherapy: a phase I/II open-label trial launched in 2005 studying urelumab in metastatic or locally advanced solid tumors (NCT00309023). Despite seeing an anti-tumor clinical response in patients, the therapeutic benefit of urelumab was dose-dependent and the drug was observed to have dose-limiting hepatotoxicity (80, 82). Utomilumab, a ligand-blocking fully human IgG2 mAb, on the other hand, demonstrated a more favorable safety profile in a phase I trial (NCT01307267) and was tolerated at higher doses while exerting relatively lower therapeutic efficacy as compared to urelumab (80, 81). Furthermore, Chin et al. observed that utomilumab required greater doses to achieve maximum activation of 4-1BB (23). Such differences in outcomes and dose requirements may, in part, be explained by the different binding properties of the two antibodies. Whereas urelumab binds to the N-terminus in CRD-1 of 4-1BB, utomilumab binds to the junction between CRD-3 and CRD-4, which is adjacent to the ligand binding site CRD2, and a part of CRD3 (23). Thus, while urelumab binds to 4-1BB independently of 4-1BBL, utomilumab binding has been shown to sterically occlude the ligand, which may explain its relatively lower efficacy. Additionally, the potent agonistic effects of urelumab could be explained by its ability to crosslink multiple receptor/ligand complexes by forming ternary complexes with 4-1BB and 4-1BBL which, in turn, enhances the activating signaling down the pathway (23). Nonetheless, due to the dose-limiting toxicities of urelumab and moderate efficacy of utomilumab, subsequent strategies largely focused on combination therapies that targeted 4-1BB along with other tumor-driving factors with the goal of drawing a potent anti-tumor response at a lower 4-1BB antibody dose, minimizing its associated adverse effects.

A common strategy studied in a variety of cancer types incorporated urelumab or utomilumab in combination with other conventional anti-tumoral therapeutics that have already been established, such as chemotherapy, radiotherapy, and other immunotherapies, with the goal of achieving synergistic or additive anti-tumor effects while minimizing unfavorable toxicities (**Table 1**). Co-treatment of the 4-1BB antibody along with different classes of chemotherapy has been shown to elicit synergistic effects by preconditioning the immune environment, enhancing the proliferation of CD8^+^ cells following 4-1BB agonism, or by inducing 4-1BB expression (83–86). Treatment with a 4-1BB antibody in addition to radiotherapy has also been observed to evoke a strengthened anti-tumor immune response when compared to monotherapy of either regimen in different preclinical tumor models (87). Such synergy was explained, in part, to be mediated by the enhanced expression of 4-1BBL following radiotherapy, which augments immune signaling down the 4-1BB/4-1BBL pathway (87). This, in turn, enabled a potent anti-tumoral response at a lower dose of the 4-1BB antibody, limiting its associated hepatotoxicity. Immune checkpoint inhibitors are also another greatly studied drug class with synergistic potential in combination with 4-1BB agonism. Commonly observed antibodies in combination with urelumab or utomilumab include those specific to the immune checkpoint receptors CTLA-4 and PD-1 as well as the ligand for PD-1, PD-L1 (**Table 1**). The agonistic effect on a costimulatory immune receptor and antagonistic effect on a coinhibitory immune receptor induced by the dual antibody treatment were observed to have synergistic anti-tumoral outcomes mediated by the enhanced effector functions and tumor infiltration of CD4^+^ and CD8^+^ T cells (88, 89). Of note, 4-1BB stimulation has been shown to be associated with upregulated PD-1 expression, whereas PD-1/PDL1 signaling functions in tumor cell resistance to 4-1BB agonism therapy (90). This could explain, in part, the synergy observed in the combination therapy of PD-1 or PD-L1 antibodies with 4-1BB targeted therapy. In addition, combination therapy of a 4-1BB antibody with a CTLA-4 antibody was shown to be capable of ameliorating the autoimmune adverse effects, including inflammation in the liver, caused by the individual antibodies in a mouse model of colon cancer, suggesting the promise of dual treatment over monotherapy (91). Apart from dual therapy with coinhibitory receptor inhibitors, 4-1BB targeted antibodies have also been studied in combination with antibodies that bind to other costimulatory TNFRs, such as OX40. Likewise, coadministration of 4-1BB and OX40 antibodies resulted in synergistic effects in the proliferation and functions of CD8^+^ T cells, produced by the concurrent stimulation of two different receptors that modulate such anti-tumor immune responses in dichotomous, independent ways (92–94).

**Table 1:** Summary of clinical trials involving urelumab or utomilumab alone or in combination with other anti-tumoral regimens.

NCT #	Drug	Conditions	Phase	Status*
NCT00309023	Urelumab	Metastatic or Locally Advanced Solid Tumors	Phase I/II	Terminated
NCT00461110	Urelumab + Chemoradiation (Radiotherapy +/- Paclitaxel and Carboplatin)	Non-Small Cell Lung Carcinoma	Phase I	Terminated
NCT00803374	Urelumab + Ipilimumab (anti-CTLA-4)	Advanced Malignant Melanoma	Phase I	Withdrawn
NCT01471210	Urelumab	Advanced and/or Metastatic Solid Tumors and Relapsed/Refractory B-cell Non-Hodgkin's Lymphoma	Phase I	Completed
NCT01775631	Urelumab + Rituximab (anti-CD20)	B-cell Non-Hodgkin’s Lymphoma	Phase I	Completed
NCT02110082	Urelumab + Cetuximab (anti-EGFR)	Advanced/Metastatic Colorectal Cancer or Advanced/Metastatic Squamous Cell Carcinoma of the Head and Neck	Phase I	Completed
NCT02252263	Urelumab + Elotuzumab (anti-CS1/ SLAMF7/CD319)	Multiple Myeloma	Phase I	Completed
NCT02253992	Urelumab + Nivolumab (anti-PD-1)	Solid Tumors and B-cell Non-Hodgkin's Lymphoma	Phase I/II	Terminated
NCT02420938	Urelumab + Rituximab (anti-CD20)	Relapsed, Refractory, or High-risk Untreated Chronic Lymphocytic Leukemia (CLL)	Phase II	Withdrawn
NCT02534506	Urelumab +/- Nivolumab (anti-PD-1)	Advanced and/or Metastatic Malignant Tumors	Phase I	Completed
NCT02652455	Urelumab + Nivolumab (anti-PD-1) + Adoptive Cell Therapy (Cyclophosphamide + Fludarabine + TIL Infusion + IL-2)	Metastatic Melanoma	Early Phase I	Active, not recruiting
NCT02845323	Urelumab + Nivolumab (anti-PD-1)	Cisplatin-Ineligible or Chemotherapy-Refusing Muscle-Invasive Urothelial Carcinoma of the Bladder	Phase II	Recruiting
NCT03431948	Urelumab + SBRT	Advanced Solid Tumors	Phase I	Active, not recruiting
NCT03792724	Urelumab + Nivolumab (anti-PD-1)	Advanced Solid Tumors	Phase I/II	Not yet recruiting
NCT01307267	Utomilumab +/- Rituximab (anti-CD20)	CD20+ Non-Hodgkin's Lymphoma	Phase I	Completed
NCT02179918	Utomilumab + MK-3475 (anti-PD-1)	Advanced Solid Tumors	Phase I	Completed
NCT02315066	Utomilumab + PF-04518600 (anti-OX40)	Advanced/Metastatic Carcinoma	Phase I	Completed
NCT02554812	Utomilumab + Avelumab (anti-PD-L1)	Locally Advanced or Metastatic Solid Tumors	Phase II	Active, not recruiting
NCT03290937	Utomilumab + Irinotecan Hydrochloride (Chemotherapy) + Cetuximab (anti-EGFR)	Metastatic Colorectal Cancer	Phase I	Active, not recruiting
NCT03217747	Utomilumab + Avelumab (anti-PD-L1) + PF-04518600 (anti-OX40)	Advanced Malignancies	Phase I/II	Active, not recruiting
NCT03258008	Utomilumab + ISA101b (Cancer Vaccine against HPV16)	HPV-16-Positive Incurable Oropharyngeal Cancer	Phase II	Completed
NCT03414658	Utomilumab + Trastuzumab (anti-HER2) + Avelumab (anti-PD-L1) +/- Vinorelbine (Chemotherapy)	Advanced HER2+ Breast Cancer	Phase II	Recruiting
NCT03318900	Utomilumab + T-Cell Infusion + Aldesleukin (IL-2)	Recurrent Ovarian Cancer	Phase I	Active, not recruiting
NCT03440567	Utomilumab + Avelumab (anti-PD-L1) + Rituximab (anti-CD20) + Chemotherapy (Carboplatin + Etoposide Phosphate + Ifosfamide) OR Utomilumab + Avelumab (anti-PD-L1) + Rituximab (anti-CD20) + Ibrutinib (TKI)	Relapsed or Refractory Diffuse Large B-Cell Lymphoma or Mantle Cell Lymphoma	Phase I	Active, not recruiting
NCT03704298	Utomilumab + Axicabtagene Ciloleucel (CAR T-cell Therapy)	Refractory Large B-cell Lymphoma	Phase I	Active, not recruiting
NCT03636503	Utomilumab + Rituximab (anti-CD20) +Avelumab (anti-PD-L1)/PF04518600 (anti-OX40)	Follicular Lymphoma	Phase I	Active, not recruiting
NCT03971409	Utomilumab + Avelumab (anti-PD-L1)	Stage IV or Unresectable, Recurrent Triple Negative Breast Cancer	Phase II	Recruiting
NCT03364348	Utomilumab + Ado-Trastuzumab Emtansine/Trastuzumab (anti-HER2)	HER2+ Advanced Breast Cancer	Phase I	Active, not recruiting

*Status as of August 2022.

CTLA-4, cytotoxic T-lymphocyte–associated antigen 4; CD20, cluster of differentiate 20; EGFR, epidermal growth factor receptor; CS1, CD2 subset-1; SLAMF7, signaling lymphocytic activation molecule family member 7; CDC319, cluster of differentiate 319; PD-1, programmed cell death-1, TIL Infusion, tumor-infiltrating lymphocyte infusion; IL-2, interleukin-2; SBRT, Stereotactic Body Radiation Therapy; PD-L1, programmed cell death ligand-1; HPV16, human papillomavirus strain 16; HER2, human epidermal growth factor receptor 2; TKI, tyrosine kinase inhibitor; CAR T-Cell Therapy, Chimeric antigen receptor T-cell therapy.

Other antibodies commonly studied in combination with 4-1BB antibodies, with the purpose of obtaining synergistic or enhanced anti-tumor responses, include those targeting epidermal growth factor receptors (EGFRs) or CD20. 4-1BB agonistic antibodies in combination with rituximab, a CD20 monoclonal antibody, have been studied in the treatment of lymphomas and leukemias where they demonstrated clinical benefit with 4-1BB stimulation on NK cells promoting their activation and expansion as well as the ADCC in response to rituximab administration (95). Similarly, 4-1BB agonism on NK cells and the resulting strengthened ADCC showed to augment the efficacy of EGFR- or human epidermal growth factor receptor 2 (HER2)- targeted antibodies, cetuximab and trastuzumab, respectively, in cancers that may have been refractory or unresponsive to monotherapy (96, 97).

**Figure 3:**
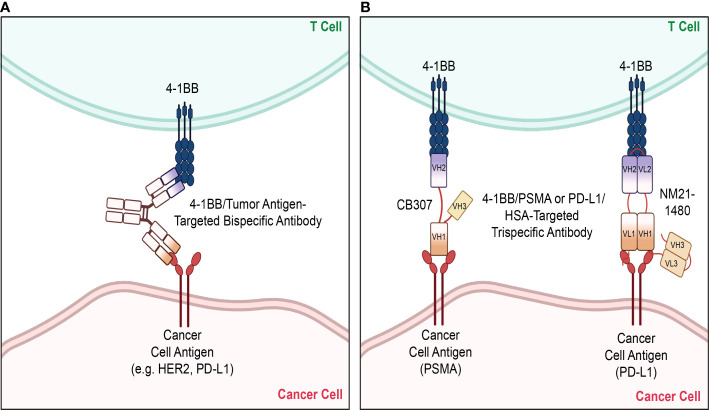
Bispecifics and Trispecifics used as therapeutic strategies. Various novel strategies have been implemented in targeting 4-1BB, with a primary focus on bispecific and trispecific antibodies. **(A)** The bispecific antibodies mainly target 4-1BB and another tumor antigen upregulated on cancer cells, such as HER2 or PD-L1, to localize and limit 4-1BB stimulation to the tumor microenvironment. **(B)** Currently studied trispecific antibodies are likewise composed of the variable regions of the individual antibodies targeting 4-1BB and the tumor antigen. A third variable region domain targeting HSA is attached to these domains, which serve the purpose of prolonging their serum half-lives. The trispecific antibody CB307 is composed of the heavy chain variable domains of the antibodies targeting 4-1BB, PSMA, and HSA. The trispecific antibody NM21-1480 is composed of both the light chain variable domains of the antibodies targeting 4-1BB, PD-L1, and HSA. HER2, human epidermal growth factor receptor 2; PD-L1, programmed cell death ligand-1; PSMA, prostate specific membrane antigen; HSA, human serum albumin, VH, heavy chain variable domain; VL, light chain variable domain. The figure was created with BioRender.com.

Apart from the conventional forms of therapy that have been discussed thus far, there is a variety of different therapeutic strategies that are being applied in combination with 4-1BB agonism to enhance therapeutic efficacy and safety. There has been an emergence in the utilization of bi- and tri- specific antibodies as well as the application of adoptive cell therapy (ACT), such as CAR T cell therapy, with the aims of enhancing tumor specific activity and reducing systemic toxicity of 4-1BB stimulation (**Tables 1**, **2**). The bi- and tri-specific antibodies commonly seen in studies are mainly composed of a 4-1BB binding fragment along with those that bind to tumor-associated receptors/antigens, primarily HER2 or PD-L1, that were discussed previously in the dual antibody regimens (**Figure 3**). Such engineering of an antibody, so that it is capable of binding to both 4-1BB and another target that is known to be commonly upregulated in and associated with tumors, sought to increase specificity of the 4-1BB activation by localizing the agonistic antibody and the consequent immune stimulation to the tumor environment. This localization preserved the synergistic anti-tumor effect seen with dual therapy while reducing the adverse effects seen when 4-1BB is stimulated nonspecifically in the peripheral tissues outside the tumor. Results from preclinical studies of 4-1BB/HER2 or 4-1BB/PD-L1 bispecific antibodies reflected the aims of this design, restricting T cell stimulation to the tumor environments that are rich in HER2 or PD-L1, respectively, and reducing the toxicity caused when 4-1BB is activated in the periphery, including in the liver (98, 99). In terms of the trispecific antibodies that are currently undergoing clinical trials (CB307, NM21-1480), like the bispecifics, they are mainly designed to target 4-1BB along with another tumor-driving or associated factor. In addition to those binding domains, they are composed of an additional, third domain that binds to human serum albumin (HSA), which serves the purpose of prolonging the serum half-life of the antibodies (100). Even more, Compte et al. have developed a bispecific 4-1BB agonistic Fc-free trimerbody composed of three 4-1BB binding single-chain variable fragments (scFv) and three EGFR binding antibodies for use in cancer treatment (101). By removing the Fc region, the authors sought to enhance the relatively lower efficacy and mitigate off-target toxicities which they attributed to full-length IgG antibodies (101–103). The different takes in the approaches in the design and development of novel, non-canonical antibodies that optimize efficacy while minimizing toxicities are suggestive of the importance and promise of 4-1BB costimulation as a therapeutic strategy.

**Table 2:** Summary of clinical trials targeting 4-1BB that do not involve urelumab or utomilumab.

NCT #	Drug	Conditions	Phase	Status*
Monotherapy				
NCT03707093	ADG106 (anti-4-1BB)	Advanced/Metastatic Solid Tumors and/or Non-Hodgkin Lymphoma	Phase I	Active, not recruiting
NCT04903873	EU101 (Anti-4-1BB)	Advanced Solid Tumors	Phase I/II	Recruiting
Combination therapy				
NCT03650348	PRS-343 + Atezolizumab (anti-PD-L1)	HER2+ Advanced or Metastatic Solid Tumors	Phase I	Active, not recruiting
NCT04077723	RO7227166 (CD19 targeted 4-1BB Ligand) + Obinutuzumab (anti-CD20) + Glofitamab (anti-CD20 and anti-CD3)	Relapsed/Refractory B-Cell Non-Hodgkin's Lymphoma	Phase I	Recruiting
NCT04121676	AGEN2373 (anti-4-1BB) +/- AGEN1181 (anti-CTLA-4)	Advanced Solid Tumors	Phase I	Recruiting
NCT04501276	ADG116 (anti-CTLA-4) + ADG 106 (anti-4-1BB)	Advanced/Metastatic Solid Tumors	Phase I	Recruiting
NCT05040932	YH004 (anti-4-1BB) +/- Toripalimab (anti-PD-1)	Advanced Solid Tumors and Relapsed or Refractory non-Hodgkin Lymphoma	Phase I	Recruiting
NCT04826003	RO7122290 (FAP-targeted 4-1BB agonist) + Cibisatamab (anti-CEA/CD3) + Obinutuzmab (anti-CD20)	Metastatic Colorectal Cancer	Phase I/II	Recruiting
NCT04694781	LVGN6051 (anti-4-1BB) +/- Pembrolizumab (anti-PD-L1)	Advanced/Metastatic Malignancy	Phase I	Recruiting
NCT04130542	LVGN6051 (anti-4-1BB) +/- Pembrolizumab (anti-PD-L1)	Advanced/Metastatic Malignancy	Phase I	Recruiting
NCT05236608	ADG106 (anti-4-1BB) + Nivolumab (anti-PD-1)	Metastatic Non-Small Cell Lung Cancer	Phase I/II	Recruiting
Bispecifics				
NCT03330561	PRS-343 (Bispecific: 4-1BB Targeting Anticalin Protein + anti-HER2)	HER2+ Advanced or Metastatic Solid Tumors	Phase I	Completed
NCT03809624	INBRX-105 (Bispecific: anti-PD-L1 + anti-41BB) +/- Pembrolizumab (anti-PD-1)	Locally Advanced or Metastatic Solid Tumors	Phase I	Recruiting
NCT04648202	FS120 (Bispecific: anti-4-1BB+ anti-OX40)	Advanced Malignancies	Phase I	Recruiting
NCT05159388	PRS-344/S095012 (Bispecific: anti-PD-L1 + anti-4-1BB - Anticalin fusion)	Advanced and/or Metastatic Solid Tumors	Phase I/II	Recruiting
NCT04762641	ABL503 (Bispecific: anti-4-1BB + anti-PD-L1)	Advanced Solid Tumors	Phase I	Recruiting
NCT04740424	FS222 (Bispecific: anti-4-1BB+ anti-PD-L1)	Advanced Malignancies	Phase I	Recruiting
NCT05190445	Cinrebafusp alfa (PRS-343) (Bispecific: anti-HER2 + anti-4-1BB-Anticalin) + Ramucirumab (anti-VEGFR2) + Paclitaxel (Chemotherapy) OR Cinrebafusp alfa (PRS-343) + Tucatinib (TKI)	HER2+ Gastric Cancer	Phase II	Recruiting
Trispecifics				
NCT04839991	CB307 (Trispecific: anti-4-1BB+ anti-PSMA + anti-HSA)	Advanced and/or Metastatic Solid Tumors	Phase I	Recruiting
NCT04442126	NM21-1480 (Trispecific: anti-PD-L1 + anti-4-1BB + anti-HSA)	Advanced Solid Tumors	Phase I/II	Recruiting

*Status as of August 2022.

HER2, human epidermal growth factor receptor 2; PD-L1, programmed cell death ligand-1; CD19, cluster of differentiate 19; CD3, cluster of differentiate 3; CTLA-4, cytotoxic T-lymphocyte–associated antigen 4; PD-1, programmed cell death-1; CEA, carcinoembryonic antigen; CD20, cluster of differentiate 20; PSMA, prostate specific membrane antigen; HSA, human serum albumin; EGFR2, Vascular endothelial growth factor receptor 2; TKI, tyrosine kinase inhibitor.

4-1BB targeting has also been studied in combination with ACTs, such as tumor infiltrating lymphocyte (TIL) infusion and CAR T cell therapy. The addition of a 4-1BB antibody to the adoptive transfer of TILs was observed to enhance TIL persistence as well as increase the effector/cytotoxic phenotype and function of CD8^+^ T cells *in vitro*, demonstrating clinical potential (104). Furthermore, in the context of CAR T cell therapy, incorporation of the 4-1BB intracellular domain into CAR T cells and signaling down the domain prevented the exhaustion of CAR T cells and prolonged their survival and expansion (105, 106). In a separate study involving a mouse model of B cell acute lymphoblastic leukemia, 4-1BB expressing CAR T cells also demonstrated enhanced anti-tumor activity with reduced adverse effects compared to CD28 expressing CAR T cells (107).

Overall, it appears that the current trend in the development of therapeutics that incorporate 4-1BB stimulating mechanisms focuses on bi- or tri-specific antibodies capable of targeting 4-1BB along with another tumor-driving factor. There is also an emergence of novel agonistic 4-1BB antibodies that are being studied in clinical trials such as ADG106 (Adagene) or EU101 (Eutilex), as monotherapy or in combination, that may perhaps have enhanced anti-tumor activities with reduced systemic toxicity as compared to urelumab or utomilumab (**Table 2**). Nonetheless, it can be inferred that future therapeutic strategies will most likely be a form of combination therapy due to the toxicity associated with nonspecific 4-1BB stimulation and limited efficacy with monotherapy. Thus, it can be expected that there will be a continuous growth of multimodal therapeutics that target a tumor-associated factor that can synergize with 4-1BB stimulation as well as localize its effects to the tumor microenvironment. Apart from such strategies, another potential tactic may be targeting the PTMs on 4-1BB. As previously discussed, the glycosylation and ubiquitination PTMs were shown to have a prominent effect on 4-1BB expression and signaling and, thus, perhaps may serve as potential targets in enhancing and prolonging 4-1BB expression on cells. In particular, Sun et al. identified the E3 ligase subunit involved in the ubiquitination and proteasomal degradation of 4-1BB, F-box/LRR-repeat protein 20 (FBXL20), which could be targeted in the development of therapeutics that enhance 4-1BB expression and signaling (52). Even more, based on the knowledge that glycosylation patterns vary in different cell types, screening for and targeting glycosylation moieties unique to cancer cells may be something that could be studied moving forward (55). All in all, it can be conjectured that future therapeutic regimens will focus and expand on the optimization of combination modalities for the treatment of different cancer types.

## Discussion

The 4-1BB receptor has long been established to be a prominent costimulatory receptor expressed on a wide variety of cell types, especially those of the immune system. This extensive expression profile, along with the role of the associated signaling pathways in harnessing a potent and durable immune response, makes the receptor an attractive target in the treatment of various diseases, including different types of cancer. The clinical potential and prominence of 4-1BB stimulation as a cancer treatment regimen is reflected in the numerous clinical trials that have been completed and are ongoing thus far. Nonetheless, the advantages of the receptor’s wide expression profile are accompanied by a critical limitation when a 4-1BB stimulating antibody is administered as monotherapy. The dose-limiting toxicities, especially hepatotoxicity, impose a limitation to the anti-tumor efficacy of the antibodies that stimulate 4-1BB. Thus, many emerging strategies sought to conserve the therapeutic potential of 4-1BB agonism while minimizing systemic toxicity in tissues outside the tumor. A strategy that has been predominantly applied in recent years and is expected to see an increase of is the utilization of bi- or tri- specific antibodies that enable the localization of 4-1BB stimulation to the tumor environment and elicit durable potent, and often synergistic, anti-tumor effects. Thus, drawing from the trends that we have summarized here, it can be projected that there will be a continuous emergence of strategies that utilize other tumor-specific targets in combination with 4-1BB agonism with the goal of localizing the immune stimulatory and anti-tumor effects of 4-1BB signaling to the tumor while minimizing off-target, peripheral 4-1BB activation. Nonetheless, there remains great room for the development and finetuning of novel strategies targeting 4-1BB as it indeed has a clinically significant and potent role in drawing durable and effective immune responses bolstered by its wide expression profile and associated signaling pathways.

## Author contributions

Conceptualization and writing, AK. The literature search, MN. Conceptualization, review, editing, and supervision, S-OL. All authors contributed to the article and approved the submitted version.

## Conflict of interest

The authors declare that the research was conducted in the absence of any commercial or financial relationships that could be construed as a potential conflict of interest.

## Publisher’s note

All claims expressed in this article are solely those of the authors and do not necessarily represent those of their affiliated organizations, or those of the publisher, the editors and the reviewers. Any product that may be evaluated in this article, or claim that may be made by its manufacturer, is not guaranteed or endorsed by the publisher.
